# An optimized antimicrobial peptide analog acts as an antibiotic adjuvant to reverse methicillin-resistant *Staphylococcus aureus*

**DOI:** 10.1038/s41538-022-00171-1

**Published:** 2022-12-12

**Authors:** Xuan Chen, Xiaoping Wu, Shaoyun Wang

**Affiliations:** 1 College of Chemical Engineering, Fuzhou University, Fuzhou, Fujian 350108 China; 2 College of Biological Science and Engineering, Fuzhou University, Fuzhou, Fujian 350108 China

**Keywords:** Antimicrobials, Peptides

## Abstract

The misuse of antibiotics in animal protein production has driven the emergence of a range of drug-resistant pathogens, which threaten existing public health security. Consequently, there is an urgent need to develop novel antimicrobials and new infection treatment options to address the challenges posed by the dramatic spread of antibiotic resistance. Piscidins, a class of fish-specific antimicrobial peptides (AMPs), are regarded as promising therapies for biomedical applications. Progress towards potential analogs from the piscidin family has been hampered by unenforceable structural optimization strategies. Here, we leverage a strategy of bioinformatics analysis combined with molecular dynamics (MD) simulation to identify specific functional hotspots in piscidins and rationally design related analogues. As expected, this approach yields a potent and non-toxic PIS-A-1 that can be used as an antibiotic adjuvant to reverse methicillin-resistant *Staphylococcus aureus* (MRSA) pathogens. Remarkably, the structural optimization scheme and application strategy proposed here will contribute richer therapeutic options for the safe production of animal protein.

## Introduction

The growing demand for animal protein is the most noteworthy dietary trend of our time. Correspondingly, the largest annual dissipation of antibiotics worldwide occurs on large-scale production of animal protein^1^. The main reason why the modern food industry is so dependent on antibiotic consumption is that antibiotics can be used as important ingredients in biocides, feed additives, and veterinary drugs to ensure the health of animal eggs, milk and meat supplied to humans^2,3^. Frighteningly, the wide application of antibiotics in animals has driven the emergence of a series of drug-resistant pathogenic and commensal microorganisms, which poses a serious threat to the existing public health security^4^. Accordingly, the development of effective antibiotic adjuvants or novel antimicrobial agents that completely replace antibiotic functions will be of great significance to ensure the safety of food raw materials and to calm the dramatic spread of antibiotic resistance.

AMPs are recognized as promising candidates because of their unique antibacterial activity and multifaceted mechanisms of action with non-specific targets^5^. For nearly 40 years after the original discovery of AMPs, the molecular mechanisms driving antimicrobial activity have been gradually revealed, mainly including the influence of various structural parameters on antimicrobial activity, such as structural propensity, amphiphilicity, hydrophobicity, and net charge^6^. The functional properties of AMPs are the result of a delicate balance of interactions between each parameter^7^. In general, adjusting a single factor to refine key parameters is the priority to obtain high antibacterial activity and low toxicity AMPs. For example, a few amino acid mutations in β-hairpin peptides can result in large changes of pore stability, pH sensitivity, potency, and selectivity for particular membrane types^8^. Consequently, traditional AMP engineering techniques based on amino acids and key parameters have been popular because of their low cost and efficiency advantages, even in the face of competition for trendy technologies including directional molecular evolution, computer-aided linguistic model design, and ancestral sequence reconstruction^9^. Nevertheless, this traditional optimization strategy has a large off-target effect and is hampered by the need to fully understand the pharmacology of candidate AMP templates^10^. So, there is an urgent need to develop another complementary toolkit to reinforce the advantages of this traditional AMP engineering technique. Indeed, AMPs are a vital component of immune defense in multicellular organisms, and their evolution over millions of years has been largely influenced by tissue-dependent host-pathogen interactions^11^. Synthetic insights into molecular evolution and population genetics of AMP genes reveal adaptive maintenance of AMP gene polymorphisms^12^. Correspondingly, certain critical AMP amino acid sequences have always been conserved during evolution, even across species^13^. For instance, chimps, gibbon, orangutans, gorillas, buffalos, and humans all express Hep-25 with the same sequence^14^. Consequently, we wondered whether the amino acid sequence information of conserved evolution could be used to guide the optimization of key parameters of AMPs.

A pair of piscidin-1 (PIS-1, FFHHIFRGIVHVGKTIHRLVTG) and piscidin-3 (PIS-3, FIHHIFRGIVHAGRSIGRFLTG) from the piscidin family of hybrid striped seabass, which are highly varied C-terminal segments and conserved N-terminal segments, were selected as good prototypes to verify this hypothesis^15^. Structurally, PIS-1 and PIS-3 have cell-penetrating properties, both of which bind to target molecules through a common structural motif but have different infiltration strengths^16^. Furthermore, it is widely believed that PIS-1 has more potential to be engineered as an effective and non-toxic antimicrobial agent than PIS-3 because PIS-1 shows stronger activity against planktonic bacteria^17,18^. However, this misconception should be corrected in time as more bactericidal advantages in PIS-3 are unearthed, such as the enormous damage to bacterial biofilms, strong binding to intracellular DNA, and low hemolysis rates to blood cells^19^. Much of our previous work has also revealed that PIS-3 is a promising candidate antimicrobial agent^16,20^. Overall, we were eager to explore a strategy for the rational design of AMPs guided by molecular evolutionary information and to obtain PIS-3 analogues with the optimal therapeutic index assisted (minimum inhibitory concentration/hemolysis rate) by this strategy. Moreover, the insights gained in this study will contribute to reducing the dependence of food production on antibiotic consumption and improving the conversion rate of AMPs in agriculture.

## Results and discussion

### Bioinformatics-guided optimization of AMP specificity

Since the elucidation of the first piscidins, multiple others have been identified from teleost fish, mainly including dicentracin, chrysophsins, epinecidin, myxinidin, gaduscidins, moronecidins, and pleurocidins^21,22^. Interestingly, the amino acid sequences of piscidin family from different fish species are highly identical, which is manifested by low C-terminal sequence homology but a highly conserved N-terminus with histidine (His), phenylalanine (Phe), and isoleucine (Ile) residues^15,23^. From an evolutionary perspective, the piscidin family may play an important role in maintaining fish survival, which reminds us that the consistency of conserved residues should be ensured when optimizing the key parameters of PIS-3^24,25^. Therefore, we retained the amino acid residues in PIS-3 that make up the G(X)4G and ATCUN backbones, which are inferred to be the structural underpinnings of the piscidin family with conformational flexibility and functional diversity^14,15,17,23^. Detailed amino acid residue information related to G(X)4G and ATCUN can be obtained from Supplementary fig. 1.

**Fig. 1:**
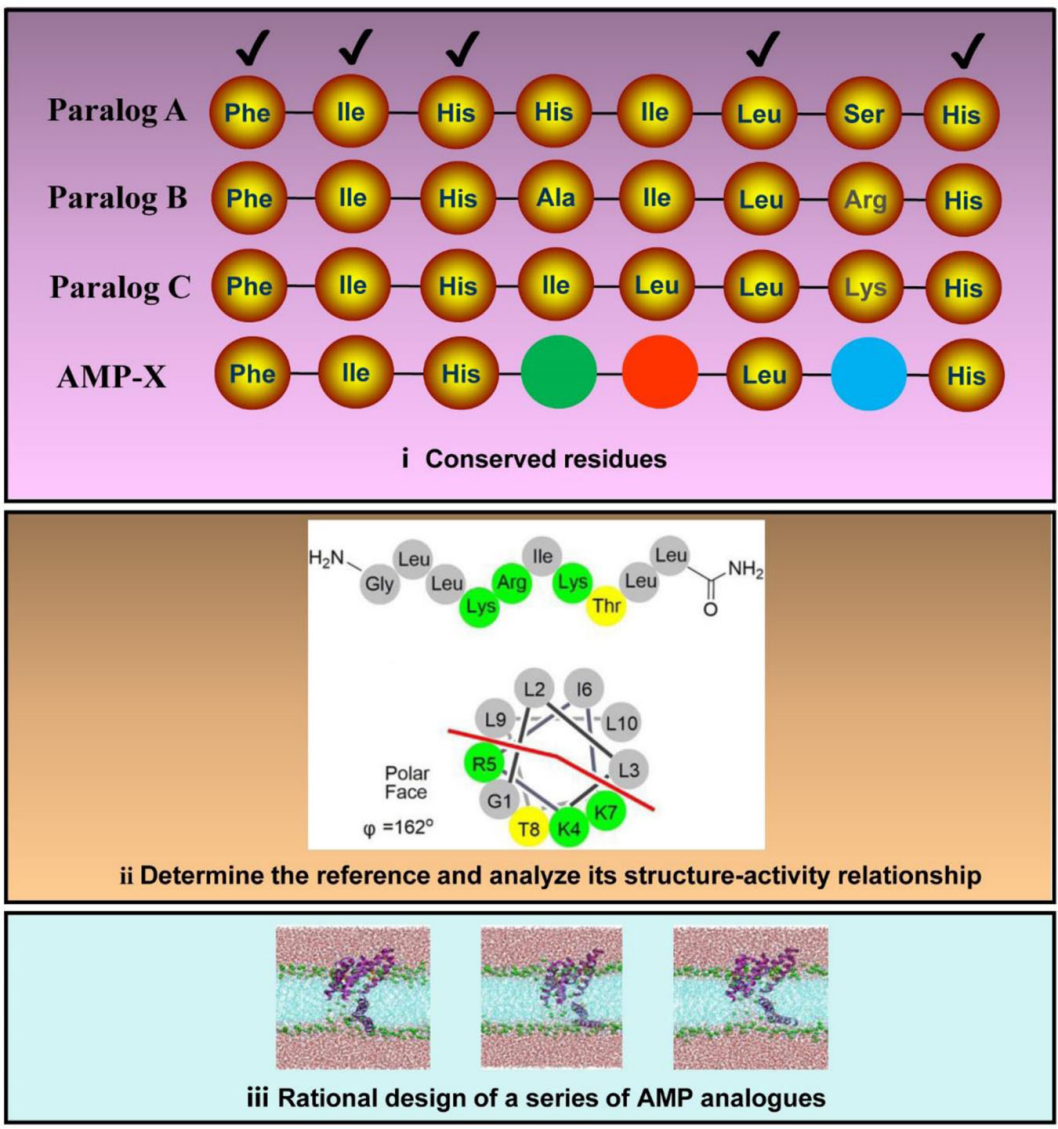
Schematic diagram of AMPs designed based on bioinformatics. First, amino acids involved in conserved structures in multiple homologous AMPs (Paralog A, Paralog B, and Paralog C) are quickly identified and retained in AMP-X. Secondly, site-directed mutations of other amino acids in AMP-X (marked with green, red, and blue spheres, respectively) are performed based on the structure-activity relationship knowledge of template AMPs. Finally, MD simulations are used to describe the interaction between AMP-X and cell membranes.

**Fig. 2:**
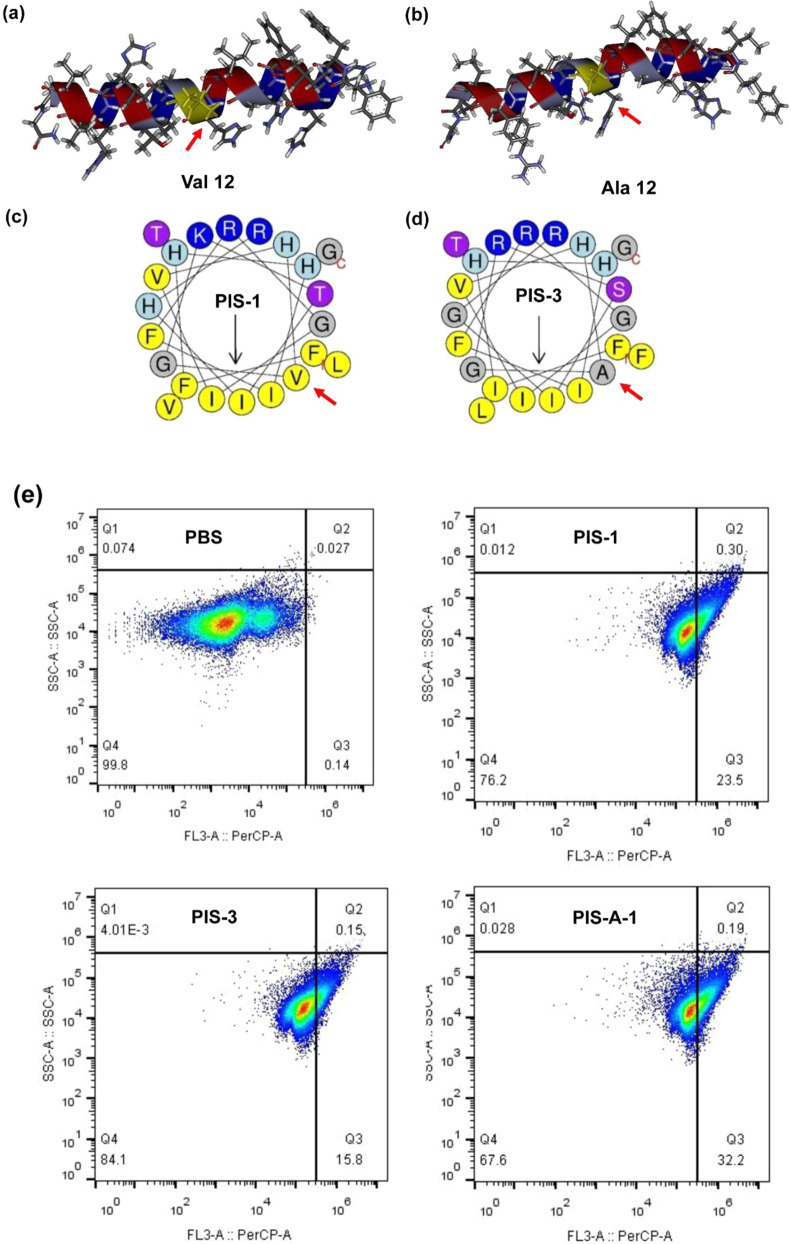
Structural analysis of PIS-1 and PIS-3. **a** Solution NMR structure of PIS-1; a ribbon representation structure of lowest energy structure with side chains labeled (blue is hydrophilic and red is hydrophobic). **b** Solution NMR structure of PIS-3. **c** Helical wheel projection of PIS-1; amino acids in dark blue are positively charged, while in dark yellow are hydrophobic. **d** Helical wheel projection of PIS-3. **e** Flow cytometric analysis.

Guided by this clue, we further replaced the key amino acid residues retained in PIS-3, which was also a directional optimization process. Notably, the determining factors for the successful implementation of this process were the identification of homologous AMPs suitable for PIS-3 comparison (Fig. 1), and the structure-activity relationship of this reference had been revealed^26^. There was no doubt that the PIS-1 is the perfect comparison for the PIS-3. Subsequently, we imitated the systematic design strategy based on reference to the bactericidal mechanism of PIS-1, where the main physicochemical properties of the PIS-3 were adjusted to achieve a tight interaction with the cytomembrane. That is, these adjustments of PIS-3 were performed by fine-tuning physicochemical functional decisive factors usually responsible for activity against bacterial membranes, such as helical propensity, charge, hydrophobic moment, and hydrophobicity (Fig. 1). Specifically, the activity of PIS-1 against planktonic bacteria was higher than that of PIS-3, which was caused by the presence of amino acid residues on the hydrophobic surface of PIS-1 that drove the AMPs to produce more defects and disturbances in the bilayer^5^. Accordingly, hydrophobic moment and hydrophobicity effects on the biological activities of PIS-3 were evaluated by replacing the original alanine (Ala 12) residues with valine (Val 12) residues and leucine (Leu 12) residues (Fig. 2a–d). Although Ala also exhibits hydrophobicity, its hydrophobicity is not as high as Val and Leu^27^. Meanwhile, the introduction of Val and Leu from the hydrophobic face into the original aliphatic residues can maintain the effects of aliphatic residues on biological function and structure. Moreover, compared with other aliphatic or aromatic hydrophobic residues, aliphatic residues Val and Leu prefer to adopt a helical structure, which has been confirmed in relation to the role of Val in PIS-1^28^. Collectively, based on the above principles (Fig. 1), we designed a series of PIS-3 analogues.

### Broad-spectrum AMP activity

The activity of the progenitor PIS-3 and newly designed derivatives was initially assessed by determining the MICs against MRSA (ATCC 43300) (Table 1). Among them, PIS-A-1 showed 4-fold increased potency compared to PIS-3 against MRSA (MIC = 8 μg/mL). To accurately determine the antimicrobial activity of PIS-A-1, MRSA cells treated with AMP were stained with PI and demonstrated by flow cytometry (Fig. 2e). PI staining of bacteria is a signal for PI to bind to cell nucleic acid, which can only occur after the membrane is destroyed^29^. The antimicrobial agents used in the test all led to extensive staining of MRSA, indicating the ability of these AMPs to destroy bacterial membranes. Bacteria treated with PIS-1 (8 μg/mL, Q3 = 23.5%) and PIS-3 (8 μg/mL, Q3 = 15.8%) to some extent showed lower levels of staining with PI than the bacteria treated with PIS-A-1 (8 μg/mL, Q3 = 32.2%), reflecting the relatively lower antibacterial activities of these peptides (Fig. 2e). Moreover, the newly designed derivatives (PIS-A-5, PIS-A-6, and PIS-A-7) composed of conserved amino acid residues from the piscidin family have no activity against MRSA, which also reflects the important role of evolutionarily conserved sequences in the function of AMPs (Table 1). Subsequently, as PIS-A-1 was the most potent antimicrobial identified among the analogues of the design, it was selected for in depth analysis. PIS-A-1 was further tested against additional Gram-negative and Gram-positive bacteria (Supplementary table 1). Compared to the original PIS-3, PIS-A-1 was highly active against both Gram-positive and Gram-negative bacteria, especially *Shigella flexneri* (CMCC 51571, MIC = 8 μg/mL) and MRSA (ATCC 43300). Interestingly, the MIC of PIS-A-1 against *Staphylococcus aureus* was similar to that of PIS-1 (MIC = 8~16 μg/mL).

**Table 1:** Minimal inhibitory concentration of PIS-3 analogues against methicillin-resistant *Staphylococcus aureus* ATCC 43300.

Peptide	Sequence	MIC (μg/mL)
PIS-1	FFHHIFRGIVHVGKTIHRLVTG	16
PIS-3	FIHHIFRGIVHAGRSIGRFLTG	32
PIS-A-1	FIHHIFRGIVHVGRSIGRFLTG	8
PIS-A-2	FIHHIFRGIVHLGRSIGRFLTG	16
PIS-A-3	FIHHIFRGIVHVGRSIG	32
PIS-A-4	FIHHIFRGIVHVGRS	32
PIS-A-5	FIHHIFRGIVHAG	≥64
PIS-A-6	GIVHAG	≥64
PIS-A-7	GIVHAGRSIGRFLTG	≥64

### Biocompatibility assessment

**Fig. 3:**
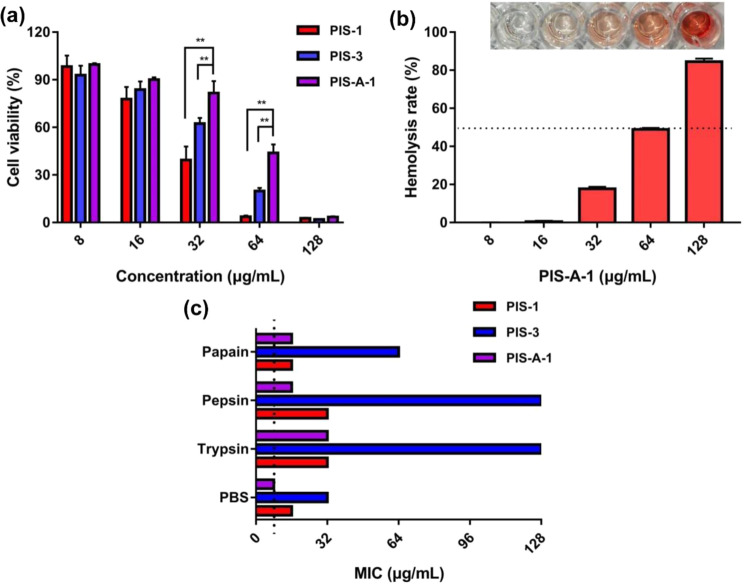
Biocompatibility evaluation of PIS-A-1. **a** Cytotoxicity of PIS-A-1 against Caco-2 cells. **b** Hemolytic activity of PIS-A-1 on mouse blood cells; 0.1% Triton X-100 or PBS is used as positive (100% hemolysis) or negative (0% hemolysis) controls. **c** Protease stability in pepsin, papain, and trypsin (final molar ratio of PIS-A-1 vs protease of 300∶1). The error bars represent standard deviation (*n* = 3).

AMPs targeting cell membranes have broad-spectrum antibacterial activity and multifaceted mechanisms of action, but also have certain cytotoxicity to normal cells. Thus, various types of cytotoxicity assays were performed using PIS-A-1 to determine whether these also showed residue sequence dependence. First, Caco-2 cells were exposed to PIS-A-1 at increasing concentrations, up to 128 μg/mL, to verify its cytotoxicity in comparison to PIS-1 and PIS-3 (Fig. 3a). In the range of 0~32 μg/mL concentration, the viability of PIS-A-1 treated cells was higher than 81.59 ± 7.56%. Even at 64 μg/mL, the cell viability was 43.87 ± 5.41%, and this is well above the concentration of antibacterial activity. In contrast, the viability of PIS-1 and PIS-3 treated cells dropped to 3.64 ± 0.79% and 19.87 ± 1.98%, respectively. Second, the hemolysis rate of PIS-A-1 was evaluated by Eq. (1) and as shown in Fig. 3b, PIS-A-1 did not induce minimal hemolysis at its MIC. Notably, this corresponds to a safe HC_50_ value, defined as the minimum AMP concentration producing 50% hemolysis of red blood cells, and the HC_50_ value of PIS-A-1 was 64 μg/mL. That is, our designed PIS-A-1 with selectivity for bacterial cells over zwitterionic mammalian cells, but it is pivotal to balance the charge and hydrophobicity to avoid undesirable increases in hemolytic activity. Finally, the stability of PIS-A-1 was assessed using enzymes with different active sites (Fig. 3c). Different enzyme treatments have resulted in an increase in the MIC of PIS-A-1 against MRSA. However, of all the AMPs here, the antimicrobial activity of PIS-A-1 was the least affected by enzymatic degradation. In other words, the ability of PIS-A-1 to resist enzymatic degradation is superior to that of PIS-3.

### PIS-A-1 structure depends on environment hydrophobicity

**Fig. 4:**
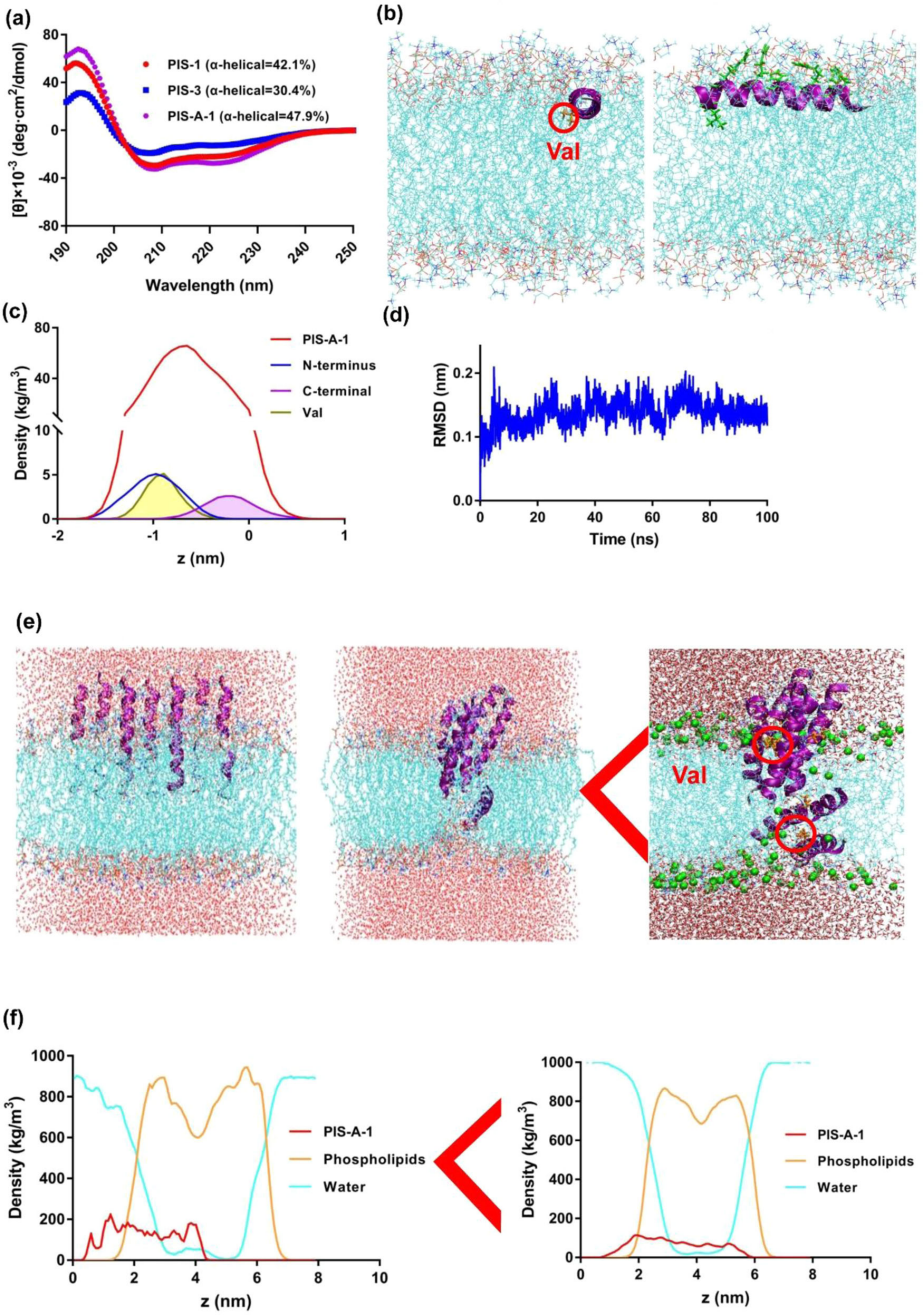
The influence of the environment on the structure of PIS-A-1. **a** CD spectrum of PIS-A-1. **b** Folded monomer inserting terminally into the membrane (top view), and folded monomer lying flat on the membrane surface (side view). Val in PIS-A-1 is marked in orange. **c** Mass density profile of folded monomer along the z-axis (normal to the membrane). **d** RMSD of folded monomer. **e** Snapshots of multiple PIS-A-1 forming transmembrane pores. **f** Mass density profile of multiple PIS-A-1 along the z-axis (normal to the membrane).

The secondary structure of AMPs (PIS-1, PIS-3, and PIS-A-1) was determined by the circular dichroism (CD) spectroscopy. The results showed that these AMPs have a similar secondary structure and exhibit typical random coil structure in aqueous solutions. Whereas, the conformation of AMPs undergoes a α-helix structure transformation in the environment of 50% TFE (Fig. 4a). The TFE here is used in CD to promote a helical structure and stabilize secondary structure^30^. In this condition, the α-helical contents of PIS-1, PIS-3, and PIS-A-1 were 42.1%, 30.4%, and 47.9%, respectively. These results suggest that mutations in the residues at key locations contribute to dramatic changes in the entire conformation of the AMP. While CD spectra indicate significant conformational differences in TFE conditions, this may not be sufficient to explain the significantly different biocompatibility and antimicrobial activity of these peptides^31^.

In order to deeply reveal the structure-activity relationship of PIS-A-1 and elucidate its mechanism of promoting membrane perturbation, MD simulation studies were carried out. MD simulation is an effective protocol for presenting atomic-level structural changes and dynamic details when AMPs interact with target membranes^32,33^. The initial adsorption mechanism of a single PIS-A-1 on the membrane surface was revealed by diffusion equilibrium simulation^34^. Figure 4b presents MD simulation snapshots at the end of the trajectory that reproduce the activity details of the individual PIS-A-1 binding and insertion of the membrane surface from different perspectives. Specifically, the interaction of single PIS-A-1 with lipid bilayers can be intuitively observed from the top-viewing, confirming the high selectivity of AMPs to pathogen membranes. This selective action is usually inferred to be the initial stage of microphase separation induced by the cationic AMPs on the multi-component membrane, through the formation of membrane inserted AMP-lipid aggregates. Interestingly, the Val residue in PIS-A-1 also interacts primarily with the hydrophobic fraction. The side view confirmed the close relationship between the hydrophilic residues on the PIS-A-1 and the head group region in the outer layer of the lipid bilayer. Furthermore, similar to the downward trend of the head group, Val residues also showed a downward trend, resulting in a good overlap of their distribution from the density profiles (Fig. 4c). The root mean square deviation (RMSD) of PIS-A-1 was less than 0.2 nm, indicating that PIS-A-1 maintained a relatively stable structure during the interaction with cell membrane (Fig. 4d). Importantly, compared with other amino acid residues, the root mean square fluctuation of Val residues was relatively higher, indicating that Val atoms contributed higher degrees of freedom when maintaining the stability of PIS-A-1 structure.

The main antibacterial mechanism of most AMPs is pore formation, which can be directly observed by simulating the dynamics and structural changes of peptides in the membrane^35^. Thus, on the basis of a single AMP system, we simulated multiple PIS-A-1. Specifically, in the snapshots of the simulation system, we found that PIS-A-1 formed transmembrane pores in the lipid bilayer and finally arranged in a V-shape (Fig. 4e). The snapshot also showed evidence of water molecules flowing through channel-like holes in the membrane. Water molecules can penetrate into the membrane through the voids between multiple PIS-A-1 hydrophilic surfaces. Moreover, the change information of the membrane thickness from 7 nm to 6 nm was obtained from the mass density profile (Fig. 4f). Among them, the thickness is determined based on the difference between the interface of the water density profile and the PIS-A-1 membrane^27^. Consequently, appropriately increasing the number of polar and basic residues in the sequence to form a larger hydrophilic channel is one of the feasible strategies to improve the antibacterial activity of PIS-A-1. Collectively, the results here suggest that the hydrophobic effect is critical for the penetration of the PIS-A-1 into the interior hydrocarbon tails of the target membrane and the formation of a stable contact between them. Crucially, the hydrophobic Val residues with shorter side chains in PIS-A-1 have significant adaptations to hydrophobic effects.

### PIS-A-1 kills antibiotic-tolerant MRSA and shows synergism with ampicillin

**Fig. 5:**
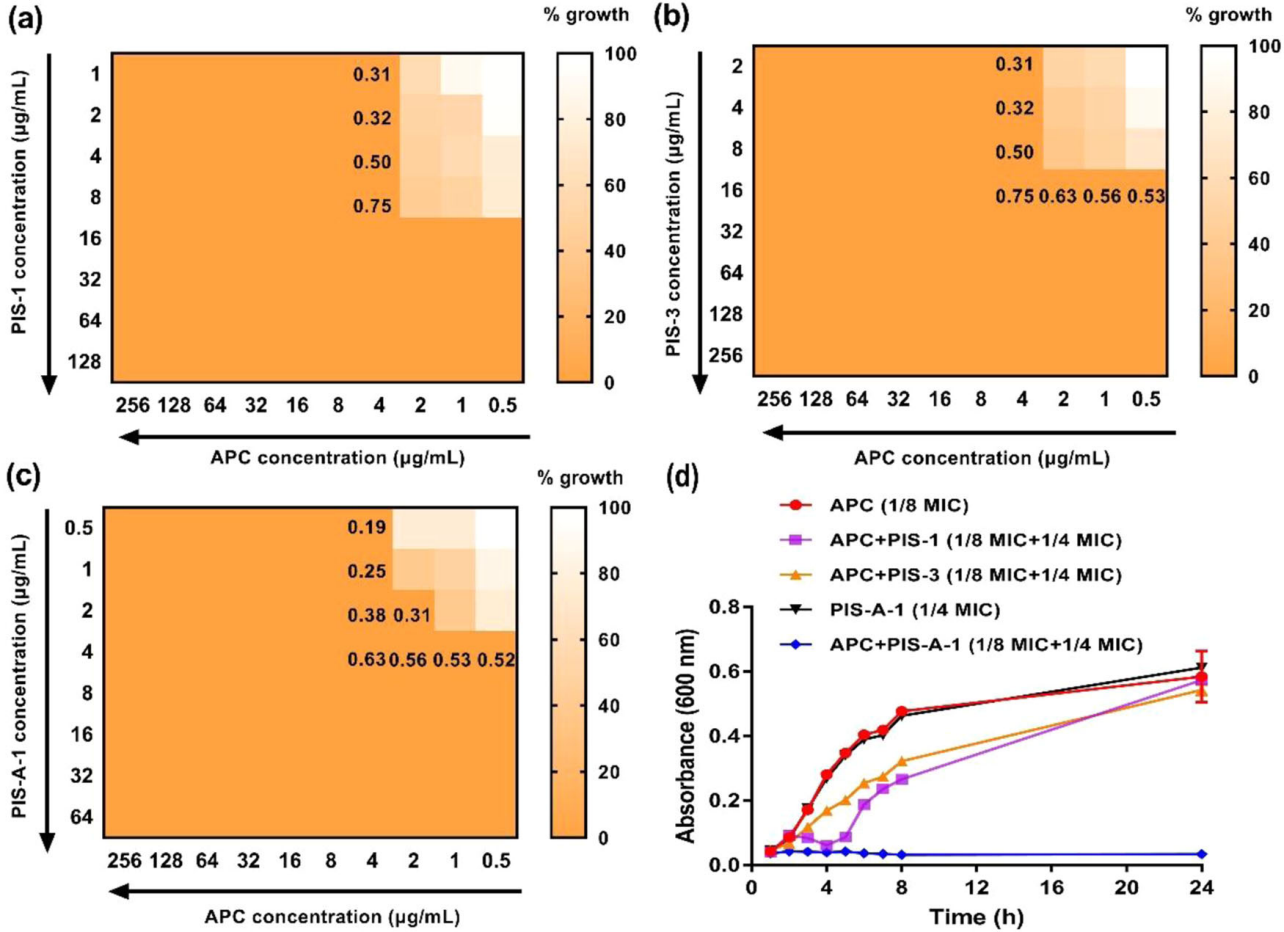
Synergistic activity between AMPs and APC against MRSA (ATCC 43300). **a**–**c** Evaluation of synergistic activity between AMPs (PIS-1, PIS-3, and PIS-A-1) and APC by chessboard assay. Dark orange regions represent higher cell density. Synergy is defined with FIC index ≤0.5. **d** Evaluation of synergistic activity between AMPs (PIS-1, PIS-3, and PIS-A-1 = 1/4 MIC) and APC (1/8 MIC) by killing kinetics.

As well known, genes encoding β-lactam-insensitive penicillin-binding protein PBP2a result in MRSA proliferating in the presence of the antibiotic APC^36^. Considering that PIS-A-1 is highly active against MRSA, we immediately explored whether PIS-A-1 can be used as an adjuvant to restore the sensitivity of MRSA to APC. Accordingly, their fractional inhibition concentration (FIC) and the corresponding FIC index are measured here by using Eq. (2)^37^. Interestingly, when tested against MRSA (ATCC 43300), we observed additive interactions between PIS-A-1 and APC. Furthermore, this additive interaction of PIS-A-1 (FIC = 0.19) was superior to PIS-1 (FIC = 0.31) and PIS-3 (FIC = 0.31), confirming the importance of Val residue mutations (Fig. 5a–c). In the growth inhibition experiment, this synergy was especially obvious, indicating that the combination of PIS-A-1 (1/4 MIC) and APC (1/8 MIC, MIC = 16 μg/mL) had strong bactericidal activity, but the two drugs had no bactericidal activity when applied separately (Fig. 5d). There are precedents for re-sensitizing MRSA to β-lactams by combination of a β-lactam with peptide antibiotics that enhance its activity, such as the synergistic effect between daptomycin and β-lactam^38^.

### Mechanistic aspects of the synergistic action

**Fig. 6:**
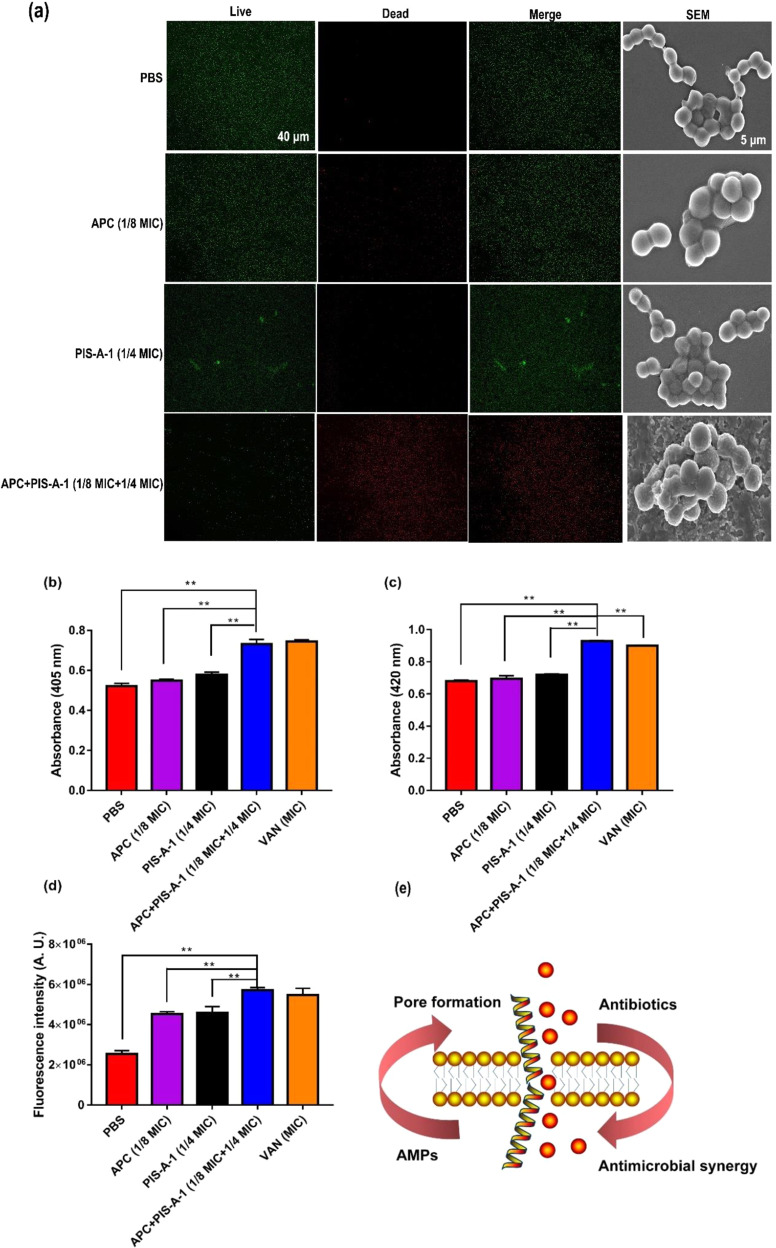
Mechanistic insights into the synergy between APC (1/8 MIC) and PIS-A-1 (1/4 MIC) against MRSA (ATCC 43300). **a** Fluorescence microscopy and SEM. Green signal: SYTO 9, red signal: PI; scale bar: 40 μm. SEM: scale bar: 5 μm. **b** The relative membrane integrity of MRSA (ATCC 43300) incubation in the presence of PBS, APC (1/8 MIC), PIS-A-1 (1/4 MIC), and APC (1/8 MIC) + PIS-A-1 (1/4 MIC) after 1 h, as determined by ONPG fluorescence. VAN (MIC = 0.25 μg/mL) is included as a positive lytic control. **c** The relative cell wall integrity of MRSA (ATCC 43300) treated with different drugs is measured by ALP kit. **d** The ROS production of MRSA (ATCC 43300) treated with different drugs is determined by ROS kit. **e** The interactions between antibiotics and AMPs constitute a positive feedback loop in which AMP-induced pore formation increases the uptake of antibiotics, which in turn, stabilizes the pore to facilitate the uptake of additional AMPs. The error bars represent standard deviation (*n* = 3).

The synergy between the two drugs may be due to the enhanced ingestion of one drug by another partner; numerous previous studies have confirmed synergies based on enhanced uptake^39–41^. When considering that the main role of PIS-A-1 is to disrupt the integrity of bacterial cell membranes, we first applied the membrane-permeable nucleic acid stain SYTO 9 and the nonmembrane-permeable nucleic acid dye propidium iodide (PI) to the fluorescence imaging analysis (Fig. 6a)^42^. The nucleic acid stains SYTO 9 and PI with divergent abilities to penetrate bacterial cells provided a two-color fluorescence background. Here, the combination of APC (1/8 MIC) and PIS-A-1(1/4 MIC) leads to an increase in the permeability of the cell membrane, which is confirmed by an increase in the number from live bacteria (green) to dead bacteria (red).

In a series of similar experiments, we further determined the disruptive effect of the APC (1/8 MIC) and PIS-A-1 (1/4 MIC) drug combinations by directly observing changes in the ultrastructure and morphology of bacterial cells (Fig. 6a). As speculated, after exposure to this drug combination, under a scanning electron microscope (SEM), the intact and smooth surface of the original cells became foamy and rough. Furthermore, perforations and fragments of certain bacterial cells were also observed here. Correspondingly, the ALP release dose of the cell group treated with the drug combination was significantly higher than that of the cell group treated with the drug alone (Fig. 6b). The release dose of ONPG contents in cell membranes also showed similar characteristics (Fig. 6c). Attentively, ALP is primarily used to evaluate cell wall breakage, whereas ONPG is applied to assess overall cell membrane breakage^42^. Moreover, under the effect of antimicrobial stressors, this may force the bacteria to produce more reactive oxygen species, stress responses may be driven, and cell membranes may change. Thus, we detected the effect of the combination group (1/8 MIC APC + 1/4 MIC PIS-A-1) on bacterial cell production ROS using the DCFDA staining method and compared it with the corresponding control group. The combination group triggered the accumulation of ROS in cells, which exacerbated membrane damage to further paralyze the homeostasis of MRSA (Fig. 6d). Taken together, these data indicated that the synergistic mechanism of APC and PIS-A-1 is associated with a deepened degree of cell membrane breakage in MRSA (ATCC 43300). That is, these results revealed that membrane perturbations caused by PIS-A-1 are a prerequisite for synergy with APC (Fig. 6e).

In this article, we performed single-factor structural optimization of the PIS-3 template based on the evolutionary implications of the piscidin family context. This was achieved by analyzing homologous residue evolutionary information in detail to rationally adjust the key parameters of PIS-3. Among them, the most promising was PIS-A-1, an AMP that tends to form α-helixes, showing broad-spectrum antimicrobial activity, high biocompatibility, and strong resistance to enzyme degradation. Meanwhile, the interaction characteristics of the designed PIS-3 analogue (PIS-A-1) with the pathogenic target were further determined by molecular dynamics simulation. The successful acquisition of PIS-A-1 implied that (i) replacement of residues at key positions on the sequence can significantly alter the biological activity of the original AMP; (ii) relatively low hydrophobic Val is more suitable than leucine or isoleucine when modified; (iii) maintaining the appropriate sequence length is essential for the biological activity of the AMP. Overall, the results here showed that evolutionary information based on homologous AMPs can be applied to PIS-3 to maximize therapeutic index while retaining membrane translocation properties, successfully generating a novel antibiotic with broad-spectrum antibacterial activity, accompanied by ameliorated hemolysis, toxicity, and enzyme stability to the progenitor. Moreover, the design of PIS-A-1 to reverse MRSA resistance against APC encourages us to discover more candidate AMPs as potential synergistic partners, and cocktail therapies containing AMPs and antibiotics are a promising strategy for revitalizing traditional antibiotics. The structure optimization scheme and application strategy of AMPs proposed here will change the current situation of massive consumption of antibiotics in food production and promote the development of sustainable agriculture.

## Methods

### Materials and reagents

Ampicillin (APC) and vancomycin (VAN) were purchased from Macklin Biochemical Co., Ltd (Shanghai, China). Mueller-Hinton broth (MHB) medium was ordered from Hope Biotechnology Co., Ltd (Qingdao, China). Pepsin, papain, and trypsin were all sourced from Aladdin (Shanghai, China). *Escherichia coli* CMCC44102, *Escherichia coli* CMCC 44817, *Escherichia coli* ATCC 8739, *Escherichia coli* CMCC 44102, *Escherichia coli* ATCC 8739, *Salmonella enterica* CMCC 50335, *Shigella flexneri* CMCC 51571, *Shigella sonnei* CMCC 51592, *Shigalla dysenteria* CMCC 51252, *Shigella flexneri* CMCC 51572, *Bacillus cereus* CMCC 63301, *Bacillus pumilus* CMCC 63202, *Staphylococcus aureus* ATCC 43300, *Staphylococcus aureus* ATCC 29213, *Staphylococcus aureus* CMCC 26003, *Staphylococcus aureus* ATCC 12600, *Staphylococcus aureus* ATCC 6538, MRSA N315, and MRSA ATCC 43300 were provided by University of Science and Technology of China. Caco-2 cells were obtained from BeNa Biotechnology Co., Ltd (Beijing, China).

### AMP synthesis

AMPs were manufactured using solid-phase peptide synthesis by GL Biochem Co., Ltd (Shanghai, China). The physicochemical properties of AMPs were evaluated using the ProtParam tool.

### Bioinformatics analysis

Amino acid sequences of the piscidin family were obtained from the National Center for Biotechnology Information (NCBI). Sequence alignment was performed using the BioEdit software. The HeliQuest program was applied to present a helix wheel projection of AMPs. Pymol 1.8 software was used to depict Three-dimensional structural models of AMPs.

### Antimicrobial activity

The minimum inhibitory concentration (MIC) was adopted to measure the antimicrobial spectrum of AMPs^42^. Specifically, experiments were executed by broth microdilution method in a 96-well plate (Corning) with an initial inoculum of 1.5 × 10^6^ Colony-Forming units (CFU) mL^−1^ in MHB based on Clinical and Laboratory Standards Institute (CLSI) guidelines. Inoculums were bacteria in the Mid-Logarithmic phase. To prevent edge effects, 96-well plates were placed in a constant temperature incubator (TSQ-280, Jianuo, China) at 37 °C. After 18 h of incubation, a microplate reader (SpectraMax i3, Molecular Devices, USA) was applied to determine the OD_600_ value of the plates. Notably, wells with no visible growth and the lowest concentration of the drug were defined as the MIC. Simultaneously, the number of inactivated bacteria treated with different drugs was quantitatively determined by flow cytometry (BD Accuri C6 Plus, USA) combined with 10 mM propidium iodide (PI, Thermo Fisher Scientific, USA)^43^.

### Cytotoxicity and hemolytic activity

Caco-2 cells cultured with RPMI 1640 medium (containing 10% FBS and 1% penicillin-streptomycin) were incubated in a humidified CO_2_ (5%) atmosphere. Next, trypsin-treated cells (0.25%) were seeded in 96-well plates at a density of 5,000 per well and incubated for 24 h. Finally, cell viability of each well with fresh medium was tested by MTT assay^44^.

Blood cells taken from mice (ICR, male, 30–35 g) were treated with different concentrations of AMPs for 1 h. Meanwhile, Triton X-100 (0.2%) and phosphate-buffered saline (PBS, 0.01 moL^−1^, pH 7.4) were used as positive and negative controls, respectively. After the reaction was completed, the OD_576_ value of each tube sample was determined and the hemolysis rate was calculated using the formula below^44,45^:

1 $${{{\mathrm{Hemolysis}}}}\,{{{\mathrm{rate}}}}\,({{{\mathrm{\% }}}}) = \left[ {\left( {{{{\mathrm{OD}}}}_{576\,{{{\mathrm{sample}}}}} - {{{\mathrm{OD}}}}_{576\,{{{\mathrm{blank}}}}}} \right)/\left( {{{{\mathrm{OD}}}}_{576\;{{{\mathrm{Triton}}}}\,{{{\mathrm{X}}}} - 100} - {{{\mathrm{OD}}}}_{576\,{{{\mathrm{blank}}}}}} \right)} \right] \times 100\%$$

### Proteolytic stability

AMPs were exposed to proteases (including pepsin, papain, and trypsin) dissolved in PBS (pH 7.4) at 37 °C for 12 h. Subsequently, proteases in AMPs were inactivated by high-temperature treatment (95 °C, 5 min) and the activity of treated AMPs against MRSA (ATCC 43300) was determined^46^. AMPs dissolved in PBS without proteases were used as controls.

### Circular dichroism

Samples with a concentration of 128 μg mL^−1^ were obtained by dissolving AMPs with water and 50% 2,2,2-Trifluoroethanol (TFE, pH 4), respectively^47^. Then, a spectrophotometer (J-1500, Jasco, Japan) was applied to determine the spectra of samples placed in a quartz cell (1 mm) from 190 nm to 250 nm. The parameters of the instrument were set to 50 nm min^−1^ scan rate, 1 nm bandwidth, and 1 s response time.

### MD simulations

Atomic MD simulations were performed using the Charmm 36 force field in the Gromacs simulation package (Version 2020.6) to describe in detail the interaction between AMPs and lipid bilayer^48^. The water molecules were modeled using the TIP3P water model^49^. The lipid bilayer system was built with 70 dioleoylphosphatidylcholine (DOPC) and 30 dioleoylphosphatidylglycerol (DOPG) on each leaflet normal to the Z-axis through the Charmm-gui input generator^50^. The initial lipid system size was around 8.7 × 8.7 × 10 nm with more than 10,000 water molecules and NaCl ions (0.15 M, 310 K) added by randomly replacing water molecules to neutralize the system charges. Simulations of the monopeptide and multi-peptide systems were performed, respectively^49^. Both systems had the protein placed on one side of the lipid bilayer before the solvation of water molecules. The systems were first equilibrated with restraints on the protein backbone for 100 ns to relax the lipid bilayer system. Then, further production runs were done for another 100 ns and the last 50 ns were used for data analysis. All systems were calculated under the NPT ensemble with semi-isotropic coupling at 1 bar on both XY and Z directions using the Nose-hoover and Parrinello-Rahman coupling methods. Nonbonding interactions were measured with an integration time-step of 2 fs and a cutoff scheme of 1.2 nm^27^. Furthermore, long-range electrostatic interactions were measured using the Particle Mesh Ewald method with a Fourier spacing of 0.1 nm. The LINCS algorithm was used to constrain all covalent bonds with hydrogen atoms^50^.

### Chequerboard studies

The interaction between AMPs and antibiotics was evaluated by a chessboard method^39,40^. The operation process of the checkerboard method was similar to the determination of MIC. Specifically, 100 μl of MHB medium was first added to each well of a 96-well plate and the antibiotic was diluted along the X-axis gradient. Gradient diluted AMPs were then added to each well along the Y-axis. Immediately, 1.5 × 10^6^ CFU mL^−1^ suspension was inoculated in this prepared plate and incubated for 18 h. Finally, the OD_600_ value of each plate was determined by microplate reader and the fractional inhibitory concentration (FIC) index was calculated by the following formula:

2 $${{{\mathrm{FIC}}}}\,{{{\mathrm{index}}}} = {{{\mathrm{MIC}}}}_{{{{\mathrm{AB}}}}}/{{{\mathrm{MIC}}}}_{{{\mathrm{A}}}} + {{{\mathrm{MIC}}}}_{{{{\mathrm{BA}}}}}/{{{\mathrm{MIC}}}}_{{{\mathrm{B}}}} = {{{\mathrm{FIC}}}}_{{{\mathrm{A}}}} + {{{\mathrm{FIC}}}}_{{{\mathrm{B}}}}$$

MIC_A_ was the MIC of drug A alone, MIC_AB_ the MIC of drug A in combination with drug B, MIC_B_ the MIC of drug B alone, MIC_BA_ the MIC of drug B in combination with drug A, and FIC_A_ was the FIC of drug A and FIC_B_ the FIC of drug B. Synergy was defined as an FIC index ≤ 0.5.

Likewise, the killing kinetics of antimicrobial agents against bacteria was tested. Briefly, 100 μL of antimicrobial agents were diluted two-fold in 96-well plates. Then, 100 μL of bacterial inoculum (1.5 × 10^6^ CFU mL^−1^) was appended and incubated at 37 °C. At a fixed time, the OD_600_ value of each well in the plate was measured by using a microplate reader (SpectraMax i3, Molecular Devices, USA).

### Electron microscopy

Bacterial cells were stained with live/dead backlight viability kits (Thermo Fisher Scientific, USA) and the overall integrity of cell membranes was observed using fluorescence microscopy (Nikon Eclipse, Japan). In general, the OD_600_ value of MRSA (ATCC 43300) in the Mid-Logarithmic phase was first adjusted to 0.2 using PBS (pH 7.4, 5000 × g, 5 min). These bacteria were then treated with drugs and dyes for 1 h and then 30 min, respectively^51^.

Scanning electron microscopy (SEM) was used to observe the status of bacterial samples. The preparation method of bacterial samples here was similar to that of a fluorescence microscope. Next, these samples were washed three times with PBS and dehydrated (10 min) in ethanol solutions (50%, 70%, 90%, and 100%) and tertiary butanol (50 and 100%). Treated samples were liquid CO_2_-dried, sputter-coated with gold-palladium, and observed using SEM equipment (Hitachi S-4800, Japan)^52^.

### Assess the permeability of cell membranes

2-Nitrophenyl-β-D-galactopyranoside (ONPG, Beyotime, Shanghai, China), alkaline phosphatase (ALP, Beyotime) and reactive oxygen species (ROS, Beyotime) assay kits were used to measure the permeability of bacterial cell membranes. Separately, drug-treated bacterial samples were washed with PBS at least 3 times and these systems were adjusted to OD_600_ = 0.5. Subsequently, 30 mmoL/L ONPG was added to these samples and the OD_405_ value was determined after 30 min incubation^42^. Likewise, the ALP content in drug-treated bacterial samples was determined according to the instructions for the kit^41^. Meanwhile, drug-treated bacterial samples were loaded with 10 μmol^−1^ of 2′,7′-dichlorofluorescein diacetate (DCFH-DA) and the ROS content in these samples was determined according to the manufacturer’s instructions^41^.

### Statistical analysis

The results of this study were statistically analyzed and presented using SPSS 19.0 and GraphPad Prism 7.0 software. All data were represented by the mean ± standard deviations. Statistical significance was analyzed using Duncan’s multiple range tests with *P* < 0.05.

### Reporting summary

Further information on research design is available in the Nature Research Reporting Summary linked to this article.

## Supplementary information

Supplementary Material

Reporting Summary

## Data Availability

We declare that all data related of this study are included in this paper and its supplementary information.
