# Supplementary material for: An optimized antimicrobial peptide analog acts as an antibiotic adjuvant to reverse methicillin-resistant Staphylococcus aureus
Source: NPJ Sci Food. 2022 Dec 12;6:57. doi: 10.1038/s41538-022-00171-1 (PMC9744894; doi:10.1038/s41538-022-00171-1)
Supplement: Supplementary file 1 — Supplementary Material [file 41538_2022_171_MOESM1_ESM.pdf]

Supplementary material:

An optimized antimicrobial peptide analog acts as an antibiotic adjuvant to reverse methicillin-resistant *Staphylococcus aureus*

Xuan Chen[1, 2], Xiaoping Wu[1, 2], Shaoyun Wang[2, *]

[1] College of Chemical Engineering, Fuzhou University, Fuzhou, Fujian 350108, China

[2] College of Biological Science and Engineering, Fuzhou University, Fuzhou, Fujian 350108, China

[*] **Corresponding author:**

Prof. Dr. Shaoyun Wang

College of Biological Science and Engineering, Fuzhou University, China

E-mail: shywang@fzu.edu.cn

Tel: +86-591-22866375. Fax: +86-591-22866278.

Supplementary table 1. Minimum inhibitory concentration of PIS-A-1

**Strains**	**PIS-1 (μ g/mL)**	**PIS-3 (μ g/mL)**	**PIS-A-1 (μ g/mL)**
**Gram-negative**			
Escherichia coli CMCC44102	16	16	16
Escherichia coli CMCC 44817	16	16	16
Escherichia coli ATCC 8739	8	8	8
Escherichia coli CMCC 44102	4	16	8
Escherichia coli ATCC 8739	8	16	8
Salmonella enterica CMCC 50335	16	16	16
Shigella flexneri CMCC 51571	8	32	8
*Sh*igella sonnei CMCC 51592	8	16	16
Shigalla Dysenteria CMCC 51252	16	32	16
Shigella flexneri CMCC 51572	8	16	16
**Gram-positive**			
Bacillus cereus CMCC 63301	16	16	8
Bacillus pumilus CMCC 63202	16	16	16
Staphylococcus aureus ATCC 43300	16	16	16
Staphylococcus aureus ATCC 29213	8	16	8
Staphylococcus aureus CMCC 26003	16	32	16
Staphylococcus aureus ATCC 12600	8	16	8
Staphylococcus aureus ATCC 6538	8	16	8
MRSA N315	8	16	8
MRSA ATCC 43300	16	32	8

Figure captions

Evolutionary interactions between host-generated AMPs. (a) Piscidin AMP alignment, showing conserved N-terminus region[1–4]. (b) Structure and function in piscidin AMP: histidine position, directionality of membrane insertion, and pH-dependent permeabilization. The AMP p1 and p3 share three histidines (H3, H4, and H11), but p1, which is significantly more permeabilizing, has a fourth histidine (H17). The permeabilization ability of p3, but not p1, is strongly inhibited at pH 6.0 when the conserved histidines are partially charged and H17 is predominantly neutral[1–4].

Supplementary figure 1

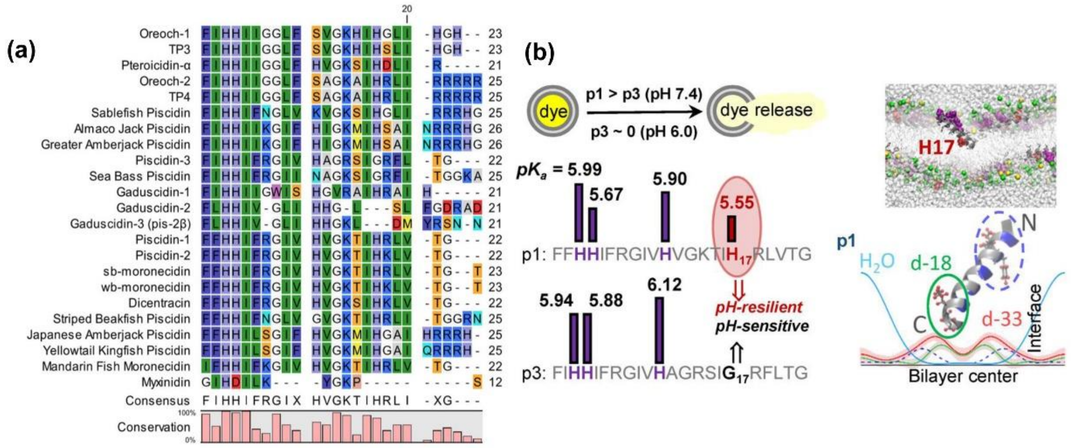

References

1. Mihailescu, M., Sorci, M., Seckute, J., Silin, V. I., Hammer, J., Perrin, B. S., Hernandez, J. I., Smajic, N., Shrestha, A., Bogardus, K. A., Greenwood, A. I., Fu, R., Blazyk, J., Pastor, R. W., Nicholson, L. K., Belfort, G. & Cotten, M. L. Structure and Function in Antimicrobial Piscidins: Histidine Position, Directionality of Membrane Insertion, and pH-Dependent Permeabilization. *J. Am. Chem. Soc.* **141,** 9837-9853 (2019).
2. Perrin, B. S., Tian, Y., Fu, R., Grant, C. V., Chekmenev, E. Y., Wieczorek, W. E., Dao, A. E., Hayden, R. M., Burzynski, C. M., Venable, R. M., Sharma, M., Opella, S. J., Pastor, R. W. & Cotten, M. L. High-Resolution Structures and Orientations of Antimicrobial Peptides Piscidin 1 and Piscidin 3 in Fluid Bilayers Reveal Tilting, Kinking, and Bilayer Immersion. *J. Am. Chem. Soc.* **136,** 3491-3504 (2014).
3. Portelinha, J., Duay, S. S., Yu, S. I., Heilemann, K., Libardo, M. D. J., Juliano, S. A., Klassen, J. L. & Angeles-Boza, A. M. Antimicrobial Peptides and Copper (II) Ions: Novel Therapeutic Opportunities. *Chem. Rev.* **121,** 2648-2712 (2021).
4. Kumar, A., Tripathi, A. K., Kathuria, M., Shree, S., Tripathi, J. K., Purshottam, R. K., Ramachandran, R., Mitra, K. & Ghosh, J. K. Single Amino Acid Substitutions at Specific Positions of the Heptad Repeat Sequence of Piscidin-1 Yielded Novel Analogs That Show Low Cytotoxicity and *In Vitro* and *In Vivo* Antiendotoxin Activity. *Antimicrob. Agents Chemother.* **60,** 3687-3699 (2016).
